# New Insights into Phosphorus Mobilisation from Sulphur-Rich Sediments: Time-Dependent Effects of Salinisation

**DOI:** 10.1371/journal.pone.0111106

**Published:** 2014-11-04

**Authors:** Josepha M. H. van Diggelen, Leon P. M. Lamers, Gijs van Dijk, Maarten J. Schaafsma, Jan G. M. Roelofs, Alfons J. P. Smolders

**Affiliations:** 1 B-WARE Research Centre, Radboud University Nijmegen, Mercator 3, Nijmegen, The Netherlands; 2 Institute for Water and Wetland Research, Department of Aquatic Ecology and Environmental Biology, Radboud University Nijmegen, Nijmegen, The Netherlands; University of Wisconsin Milwaukee, United States of America

## Abstract

Internal phosphorus (P) mobilisation from aquatic sediments is an important process adding to eutrophication problems in wetlands. Salinisation, a fast growing global problem, is thought to affect P behaviour. Although several studies have addressed the effects of salinisation, interactions between salinity changes and nutrient cycling in freshwater systems are not fully understood. To tackle eutrophication, a clear understanding of the interacting effects of sediment characteristics and surface water quality is vital. In the present study, P release from two eutrophic sediments, both characterized by high pore water P and very low pore water iron (Fe^2+^) concentrations, was studied in a long-term aquarium experiment, using three salinity levels. Sediment P release was expected to be mainly driven by diffusion, due to the eutrophic conditions and low iron availability. Unexpectedly, this only seemed to be the driving mechanism in the short term (0–10 weeks). In the long term (>80 weeks), P mobilisation was absent in most treatments. This can most likely be explained by the oxidation of the sediment-water interface where Fe^2+^ immobilises P, even though it is commonly assumed that free Fe^2+^ concentrations need to be higher for this. Therefore, a controlling mechanism is suggested in which the partial oxidation of iron-sulphides in the sediment plays a key role, releasing extra Fe^2+^ at the sediment-water interface. Although salinisation was shown to lower short-term P mobilisation as a result of increased calcium concentrations, it may increase long-term P mobilisation by the interactions between sulphate reduction and oxygen availability. Our study showed time-dependent responses of sediment P mobilisation in relation to salinity, suggesting that sulphur plays an important role in the release of P from FeS_x_-rich sediments, its biogeochemical effect depending on the availability of Fe^2+^ and O_2_.

## Introduction

The eutrophication of surface waters is an urgent problem worldwide [1]. Increased P concentrations have led to a strong decline of the biodiversity in freshwater wetlands, due to the resulting dominance of highly competitive macrophytes, and of algae and cyanobacteria, monopolising light [1]–[3]. Salinisation of freshwater systems has received increasing attention, especially in relation to climate change and sea level rise [4]. With increasing salinity, higher P concentrations are often found in surface waters (e.g. [5]–[8]), which may affect P cycling in freshwater systems. Therefore, salinisation is expected to enhance eutrophication in coastal, freshwater wetlands, leading to water quality deterioration and loss of biodiversity.

Internal mobilisation of P from eutrophic aquatic sediments is an important process adding to eutrophication problems in wetlands [9]–[12]. The classic theoretical framework suggests that sufficiently high oxygen (O_2_) concentrations in the surface water can prevent P release from the sediment [13]–[14]. According to this, the oxidation of dissolved iron (Fe^2+^) in the sediment will result in the formation of iron oxides and hydroxides (Fe(OH)_x_) at the sediment surface, effectively binding P and thereby preventing its release to the surface water. Under anaerobic conditions, these ferric compounds will be mobilised by Fe-reducing bacteria, and part of the P is released to the surface water.

Besides anaerobic conditions, increased sulphate (SO_4_^2-^) reduction rates are also known to be able to increase P mobilisation by decoupling Fe - P interactions at the sediment-water interface [12], [15]–[19]. Sulphide (S^2-^) binds efficiently to dissolved Fe^2+^ in sediment pore water, and most Fe^2+^ can become bound as iron sulphides (FeS_x_) in the sediment, strongly decreasing Fe^2+^ sediment pore water concentrations [18], [19]. Geurts *et al.* [19] found that, in aerobic surface waters, P mobilisation from sediments with low pore water Fe:P ratios (<1 mol mol^-1^) was a linear function of sediment pore water P concentrations. As a result, one would expect a release of P irrespective of the O_2_ concentration in the surface water of SO_4_^2-^ enriched wetlands [10], [12], [18], [20]. In addition, dissolved P concentrations might further increase due to the enhanced anaerobic breakdown of organic matter linked to SO_4_^2-^ reduction and concomitant mineralisation of P [2], [12], [19].

Salinisation of freshwater systems can enhance SO_4_^2-^ reduction rates due to a higher SO_4_^2-^ availability [21], which may strongly affect P mobilisation as described above. Moreover, increasing Cl^-^ and SO_4_^2-^ concentrations might enhance P release from sediments by competition for anion binding sites [10], [22]. At the same time, an increase in salinity also leads to increased Ca^2+^ concentrations [21], which may result in the immobilisation of P by co-precipitation with Ca^2+^ and calcium carbonate (CaCO_3_) [4], [9], [23]. Salinity changes affect a suite of biogeochemical processes in freshwater systems, where the net effect on P mobilisation is the combined result of these processes. Moreover, a time-dependent shift in dominance of each process on P release can be expected [18]. Most studies regarding P release focus on relative short-term effects ranging from one day to 90 days [6], [19], [20], [24], while long-term experiments are mostly lacking. In this paper we explore the time-dependent release of P from eutrophic sediments under different salinities, which is highly relevant regarding the worldwide interest in salinisation effects on freshwater wetland functioning.

To test time-dependent interactions between salinisation and P mobilisation, a controlled aquarium experiment was set up that lasted two years. Two FeS_x_-rich sediments from a coastal freshwater wetland were subjected to three naturally occurring water types characterised by different salinities. Pore waters of the peat sediments were typically rich in P and S, and very poor in Fe, and the low total Fe:S ratios in the sediment suggested that most Fe was bound to reduced S [2]. In such sediments, a very high release of P from the sediment to the surface water can be expected, predominantly depending on pore water P concentrations [10], [12], [18]–[20]. By monitoring biogeochemical changes in porewater and surface water under controlled conditions, we try to reveal how salinity affects short-term and long-term P release, in these type of sediments common for coastal wetlands.

## Materials and Methods

### 2.1 Sampling area

In this study, peat sediments were used from the coastal lowland fen area Wormer- and Jisperveld (52° 30′ 42.7644″; 4° 52′ 27.3756″) in the Netherlands. Due to historic intrusion of brackish water, peat rich in minerals such as S, Ca and Fe has accumulated in this area. After more than 50 years of desalinisation resulting from altered hydrological conditions, it gradually became a freshwater system. The peatland comprises ca. 500 ha of open water and ca. 1660 ha of peat meadows, predominantly used for agricultural purposes and partly for nature conservation. Drainage is a standard procedure in this area, leading to peat decomposition and land subsidence. As a result, risks of flooding events and salinisation are increasing in this freshwater peatland.

### 2.2 Experimental design

On 18 March 2008, two types of submerged peat sediment were collected from a ditch at a depth of 0–20 cm (ca. 25 L in total), using a sediment multi sampler (Eijkelkamp Agrisearch Equipment). Although both sediments were relatively rich in organic S and P, they differed in P availability (sediment characteristics are given in Table 1). To minimise O_2_ intrusion, the sediments were stored anaerobically at 4°C in large, closed containers. The next day, 12 glass cylinders (diameter 15 cm, height 60 cm) were filled with 15 cm of sediment A and another 12 cylinders with 15 cm of sediment B. Next 40 cm of water was carefully poured on top of the sediments, avoiding re-suspension of sediment particles. Artificially composed surface water, based on site conditions (control treatment, Table 2), was used for all sediments during an acclimatisation period of 4 weeks. The experiment was carried out in the dark at a constant and environmentally relevant temperature of 15°C. To allow oxygen diffusion to the surface water, an open cylinder system was used.

**Table 1:** Characteristics of the two sediments used.

		Organic	Bulk	Total amounts bound to sediment							
Sediment		content	Density	Total - P	Org - P	Inorg - P	Total - Al	Total - Ca	Total - S	Total - Fe	Fe:S ratio
		%	kg DW L^−1^ FW	mmol L^−1^ FW	mmol L^−1^ FW	mmol L^−1^ FW	mmol L^−1^ FW	mmol L^−1^ FW	mmol L^−1^ FW	mmol L^−1^ FW	mol mol^−1^
**A**	**Mean**	**59.7**	**0.17**	**2.9^a^**	**1.8**	**1.2^a^**	**44.5**	**54.9**	**99.3**	**33.1**	**0.33^a^**
	SEM	4.8	0.03	0.4	0.3	0.1	12.6	5.4	8.1	3.9	0.024
**B**	**Mean**	**59.6**	**0.13**	**4.7^b^**	**2.4**	**2.3^b^**	**43.4**	**44.0**	**83.6**	**38.3**	**0.46^b^**
	SEM	7.4	0.02	0.3	0.1	0.1	4.4	0.8	4.7	2.3	0.001

Significant differences between the sediment types are indicated by different letters.

**Table 2:** Chemical composition of the surface water used for the different treatments (low, normal or high salinity).

	Low salinity	Normal salinity	High salinity
	Rain water	Fresh water	Brackish water
Element	µmol L^−1^	µmol L^−1^	µmol L^−1^
Na^+^	100	7000	85000
Cl^-^	100	7000	85000
SO_4_^2-^	5	1500	5500
K^+^	30	500	1000
Ca^2+^	10	2000	2500
Mg^2+^	10	1250	3750
HCO_3_^-^	0	4000	4000
NO_3_^-^	50	50	50
NH_4_^+^	50	50	50

After this acclimatisation period, three different surface water types were applied as salinity treatments: rainwater (low salinity; 100 µmol Cl L^−1^), brackish water (high salinity; 85 mmol Cl L^−1^) and freshwater (control; 7 mmol Cl L^−1^). All treatments were artificially composed, based on field measurements (Table 2). Control water simulated water quality in the current conditions that exist in the wetland, brackish water composition was based on the historic conditions reported by Reigersman in 1946 [25]. Rainwater quality equalled the chemical composition of atmospheric deposition as measured in the Netherlands [26]. No P was added in order to be able to estimate the release of P from the sediment. For each treatment and sediment type, 4 replicates were used (24 cylinders in total).

Treatment solutions were stored in polyethylene containers (10 L), from which they were pumped into the cylinders using Masterflex L/S multichannel pumps (model 7535-08). The treatments were started by replacing the control water with the appropriate treatment water during 4 weeks, to ensure that all treatment solutions were added properly. Directly after treatment addition (week 10), stagnant conditions were created in order to measure short-term effects of P and S release from the sediment. Short-term mobilisation rates were calculated from the linear increase of the surface water P and S concentrations (0–10 weeks of the stagnant period). After a stagnant period of 26 weeks, pumps were running with a hydraulic retention time of 25 weeks for the treatment solutions during 48 weeks in order to maintain the appropriate treatment conditions. To measure long-term effects of salinity changes on the release of P and S from the sediment, pumps were stopped again (week 81) to create another stagnant period for 32 weeks. Long-term mobilisation rates were again calculated from the linear increase of the S and P concentrations in the surface water during this stagnant period.

Intact peat cores from the same location as the main experiment were collected separately, to test the effects of aerobic versus anaerobic conditions of the surface water on P release. Water and sediment oxygen (O_2_) profiles were measured, using a fixed fiber optical oxygen microsensor (optode) in combination with a Microx TX3 transmitter (PreSens Precision Sensing GmbH). The peat sediment cores were monitored during 18 weeks of either aerobic conditions similar to those of the main experiment, or anaerobic conditions by gently supplying N_2_ to the surface water. During both aerobic and anaerobic conditions, P mobilisation rates were calculated from the linear increase in surface water P concentrations.

### 2.3 Chemical analyses

To monitor water quality, samples of surface water and pore water were collected every 2 months and analysed during the experiment. Pore water was collected anaerobically, using 30 mL vacuum bottles connected to Rhizon SMS-10 cm samplers that were fixed in the upper 10 cm of the sediment (Eijkelkamp Agrisearch Equipment). Disturbance of the sediment and water was minimised by the low frequency of sampling and small sample sizes (max. 25 mL). Sulphide concentrations were determined directly after the collection by fixing 10.5 mL pore water with 10.5 mL Sulphide Anti Oxidant Buffer (SAOB), and using an Orion sulphide-electrode and a Consort Ion meter (type C830) [27]. The pH and alkalinity of all samples were measured within 24 hours after sampling, using a combined pH electrode (Radiometer) in combination with a TIM840 pH meter and a Titration Manager Titralab Autoburette. Dissolved total inorganic carbon (TIC) was measured within 24 hours after sampling by injecting 0.2 mL pore water or surface water in a closed chamber containing 0.2 M H_3_PO_4_ solution, converting all dissolved TIC into CO_2_. A continues gas flow (N_2_) directly transports the CO_2_ to an ABB Advance optima Infrared Gas Analyzer (IRGA) to measure total inorganic C concentrations. A calibration curve was made by injecting different volumes (0.1–1.0 mL) of 1.25 mM HCO_3_^-^ solution. Prior to storage at 4°C until elemental analysis, 0.1 mL HNO_3_^-^ (65%) was added to 10 mL of each sample to prevent metal precipitation. Concentrations of dissolved Ca, Fe, P, S, and Al in these stored samples were measured using an Inductively Coupled Plasma Spectrophotometer (ICP IRIS Intrepid ΙΙ XDL; Thermo Electron Corporation). Due to the anaerobic sampling of pore water, measured Fe predominantly consisted of dissolved Fe^2+^ rather than far less mobile Fe^3+^. The remaining samples were stored at −20°C in order to determine the following ion concentrations colourimetrically on Auto Analyzer 3 systems (Bran and Luebbe): NO_3_^-^ [28], NH_4_^+^ [29], ortho-PO_4_^3-^ [30] and Cl^-^ [31]. Na^+^ and K^+^ were determined with a Technicon Flame Photometer IV Control (Technicon Corporation).

For both sediments gravimetric water contents were determined by drying for 48 h at 70°C. Organic matter contents were estimated by loss on ignition for 4 h at 550°C. A homogenized portion of 200 mg dry sediment was digested in 5 mL HNO_3_ (65%) and 2 mL H_2_O_2_ (30%), using an Ethos 1 Advanced microwave digestion system (Milestone Inc.). Digestates were diluted and analysed by ICP as described above. In order to distinguish between the organic and inorganic P fraction, a P-fractionation procedure was carried out adapted after Golterman [32].

### 2.4 Statistical analyses

For statistical analysis, SPSS Statistics for Windows (Version 21.0. IBM Corp. Armonk, NY; 2012) was used. To test for differences among treatments in sediment analyses (single measurements) or differences in calculated mobilisation rates, the General Linear Model (GLM) univariate procedure combined with Tukey's-b post-hoc test was used.

To test for significant differences among treatments in repeated measurements, a GLM mixed model procedure was used. When significant differences between the two sediments were found, using a 2-way GLM mixed model with treatment as fixed factor, sediment as random factor and time as repeated measures, both sediments were analysed separately. In this separate model for sediments, time was used as repeated measures and treatment as fixed factor, with AR(1) heterogeneous as the covariance type. A Bonferroni post-hoc test was used to test for differences between treatments.

### 2.5 Ethics statement

This study was part of the National Research Programme ‘Wormer- en Jisperwater’, funded by the Dutch Ministry of Agriculture, Nature and Food Quality (LNV), within the framework of ‘Nota Ruimte’. The water management authority ‘Hoogheemraadschap Hollands Noorderkwartier’ facilitated this programme and the nature management authority ‘Natuurmonumenten’ gave permission to take samples in their reserve.

## Results

### 3.1 Pore water chemistry

**Figure 1:**
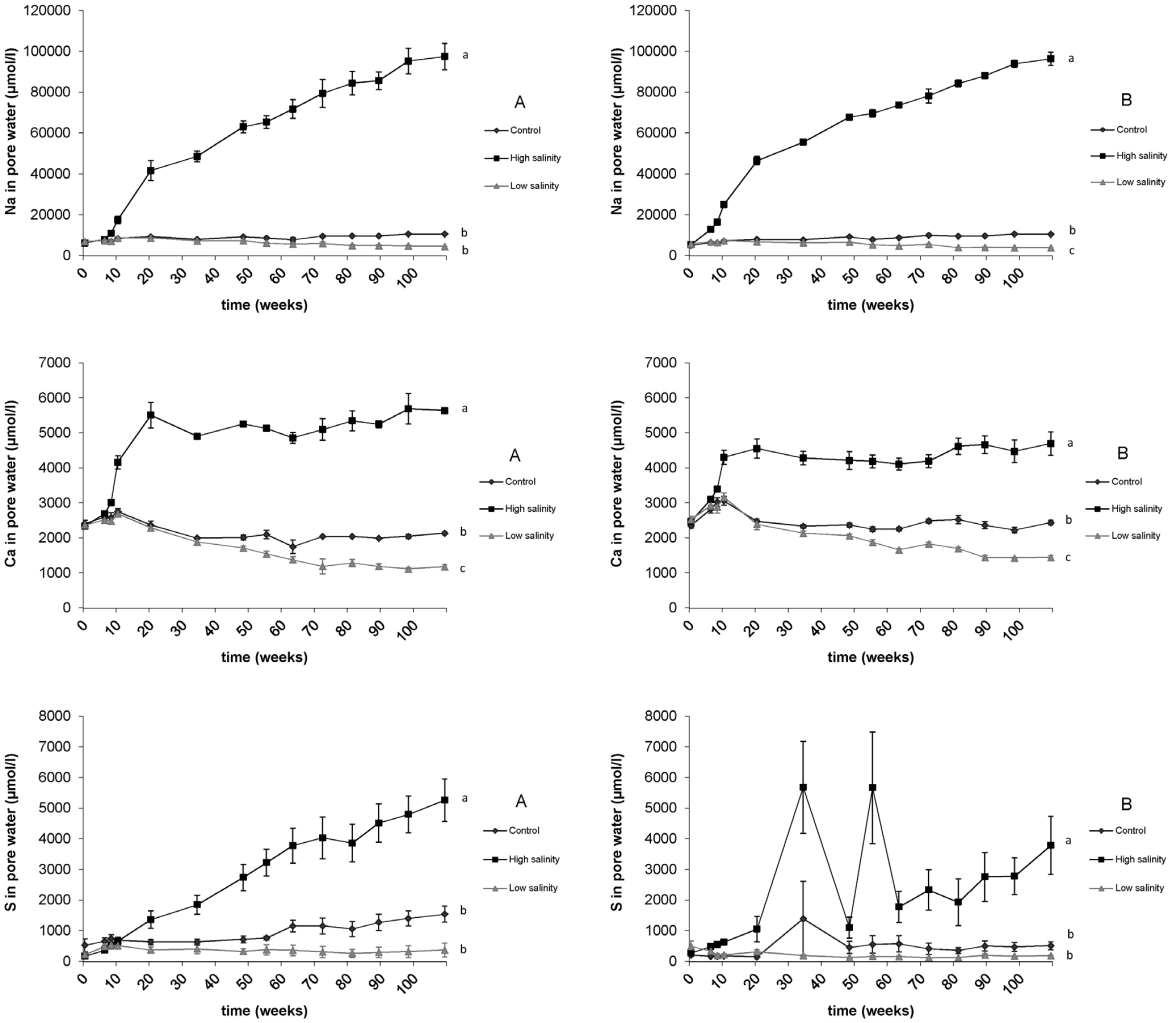
Sodium (Na^+^), calcium (Ca^2+^) and sulphur (S) pore water concentrations (µmol L^−1^) in both sediments (A: left, B: right). Significant differences between treatments are indicated with different letters.

As expected, pore water chemistry was strongly affected by changes in surface water salinity (Fig. 1). Under brackish conditions, Na^+^ showed a highly significant (p<0.005) gradual increase in the pore water over time. For the low salinity and control treatment, no significant changes in pore water Na^+^ concentrations occurred in sediment A, while Na^+^ concentrations showed a significant decrease (p<0.05) over time at a low salinity in sediment B. An interaction between treatment and sediment type was found for both Na^+^ and S concentrations, which means that the treatments had a significant, but different, effect on the two sediments. Pore water S concentrations also showed a highly significant (p<0.005) gradual increase at the high salinity treatment in both sediments (Fig. 1). At a low salinity, S concentrations remained at a steady level while the control treatment showed a small, but not significant, increase. Moreover, no clear differences in sulphide concentrations were found between sediments or treatments (average values ranged between 0–50 µmol L^−1^ for sediment A, and between 0–500 µmol L^−1^ for sediment B; data not shown).

As a result of a higher salinity, Ca^2+^ was mobilised in the sediment, as shown by significantly (p<0.005) increased pore water Ca^2+^ concentrations (Fig. 1). This increase in the pore water, well above the added concentration of 2500 µmol L^−1^ to the surface water, started directly after the onset of the high salinity treatment and Ca^2+^ concentrations remained at a steady high level during the course of the experiment.

**Figure 2:**
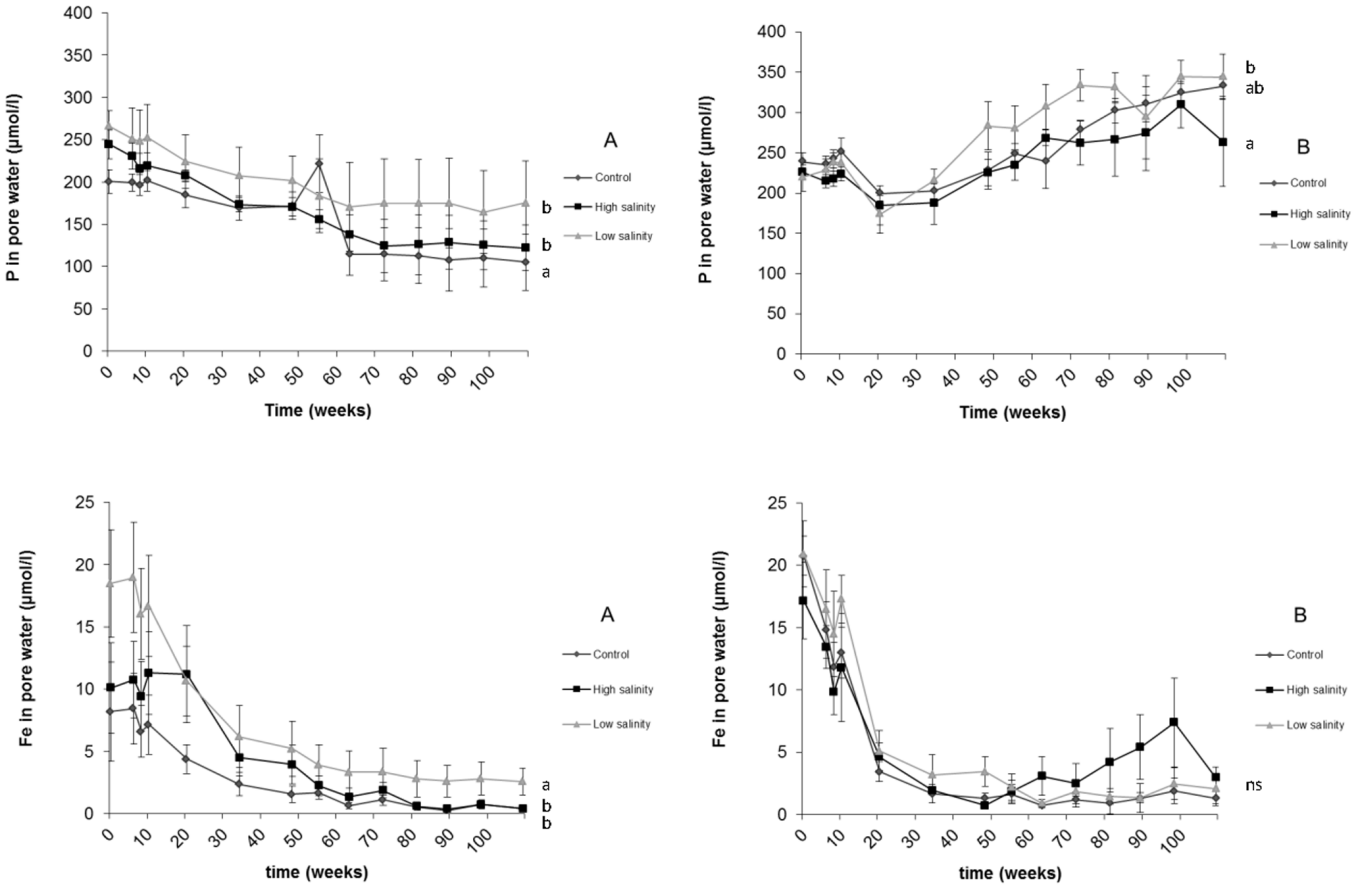
Phosphorus (P) and iron (Fe^2+^) concentrations (µmol L^−1^) in pore water of both sediments (A: left, B: right). Significant differences between treatments are indicated with different letters.

Dissolved Fe^2+^ concentrations were low and showed a gradual decrease for all treatments over time in both sediment types (Fig. 2). A significantly higher (p<0.005) Fe^2+^ concentration was found for the low salinity treatment at sediment A when compared to the control and higher salinity treatment. In contrast, no differences in pore water Fe^2+^ concentrations between treatments were found for sediment B. Pore water HCO_3_^-^ concentrations were significantly higher (p<0.05) under brackish conditions at sediment B, while no differences were found between treatments at sediment A (data not shown).

Pore water P concentrations showed a gradual decrease at sediment A for all treatments. Moreover, a significantly (p<0.05) stronger decrease of P in pore water was found for the control treatment, compared to the high and low salinity treatment at sediment A. In strong contrast, P concentrations showed a gradual increase in the pore water of sediment B for all salinity treatments (Fig. 2), with the significantly (p<0.05) lowest P concentrations in the high salinity treatment.

### 3.2 Surface water chemistry

**Figure 3:**
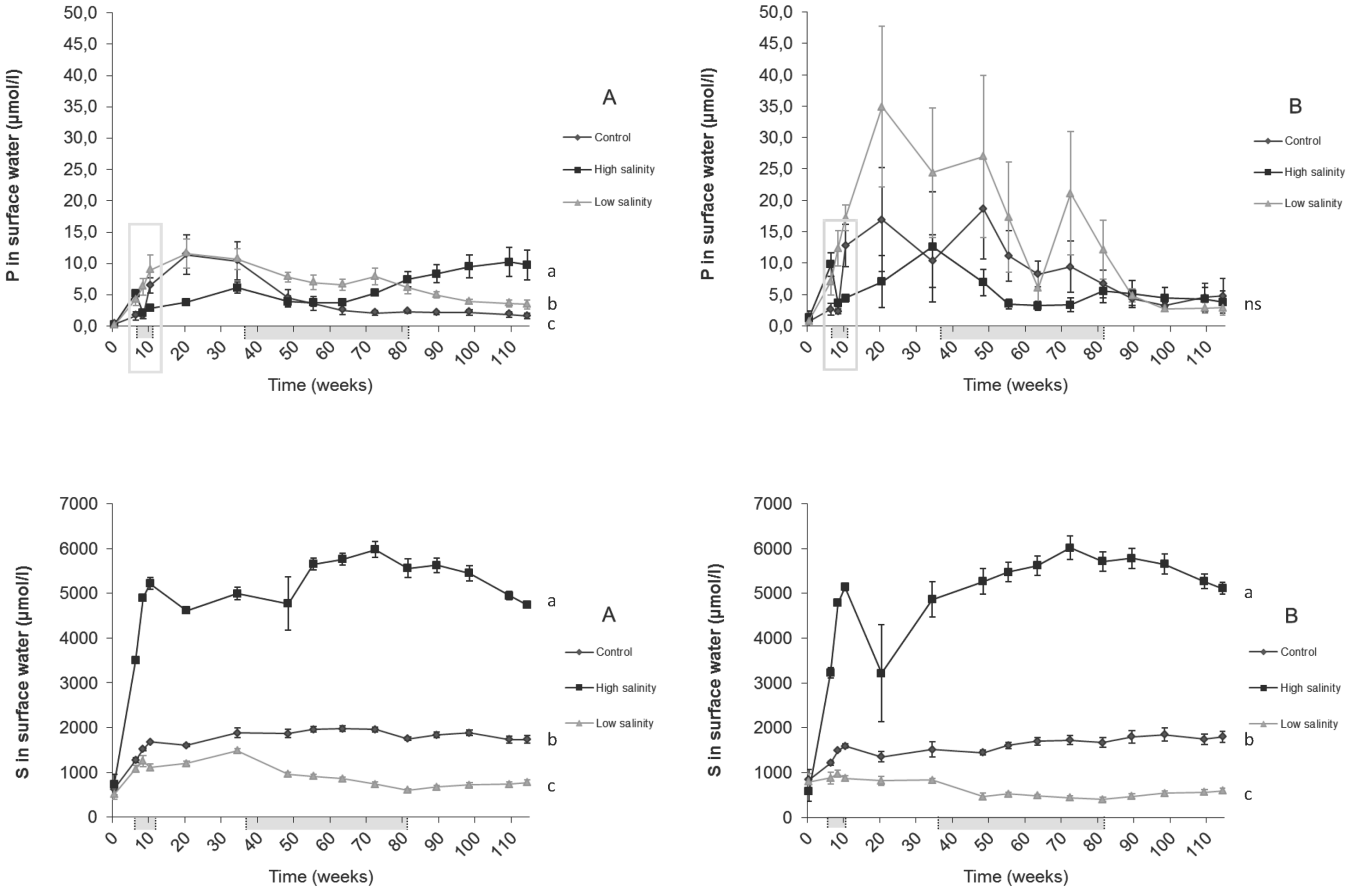
Phosphorus (P) and sulphur (S) concentrations (µmol L^−1^) in the surface water above both sediments (A: left, B: right). Significant differences between treatments are indicated with different letters. Grey shadings under the x-axis indicate periods with through-flow (see Materials and Methods).

A higher salinity led to gradually increased Na^+^ and S concentrations in the surface water, and showed significant (p<0.005) differences among all treatments, which eventually equalled the concentrations added (S: Fig. 3; Na^+^: data not shown). For the low salinity treatment, however, S concentrations in the surface water reached much higher concentrations than the concentrations of the treatment water, which suggests S mobilisation from the sediment. These S mobilisation rates were calculated (Table 3) for both a short term, showing significantly (p<0.005) negative rates at a high salinity (high S consumption) for sediment A, and for a long term, still showing significantly (p<0.005) negative S mobilisation rates at a high salinity in both sediments. In the surface water, Ca^2+^ concentrations also increased and differed significantly (p<0.005) among all treatments for both sediments (data not shown). However, both the low and high salinity treatment led to much higher concentrations than the added concentrations.

**Table 3:** P and S mobilisation rates (µmol m^−2^ day^−1^) during stagnant conditions in the short term (0–10 weeks) and in the long term (80–110 weeks).

			P mobilisation (µmol m^−2^ day^−1^)		S mobilisation (µmol m^−2^ day^−1^)	
			short term	long term	short term	long term
A	High salinity	**Mean**	**6.8**	**4.1 ^a^**	**−1916.4 ^a^**	**−1447.5 ^a^**
		SEM	1.6	2.4	86.7	243.0
	Control	**Mean**	**37.7**	**−1.1 ^ab^**	**140.9 ^b^**	**−87.0 ^b^**
		SEM	14.3	0.6	216.0	86.4
	Low salinity	**Mean**	**19.8**	**−4.0 ^b^**	**−6.5 ^b^**	**244.2 ^b^**
		SEM	12.9	2.3	425.0	69.2
B	High salinity	**Mean**	**16.3**	**−2.9**	**−8536.5**	**−1121.7 ^a^**
		SEM	24.2	1.0	5474.0	141.3
	Control	**Mean**	**53.3**	**−2.2**	**−840.4**	**123.6 ^b^**
		SEM	36.6	2.5	517.8	91.1
	Low salinity	**Mean**	**102.6**	**−13.1**	**−597.4**	**294.8 ^b^**
		SEM	74.3	6.5	236.3	83.3

Significant differences between treatments are indicated by different letters.

In the low salinity and control treatment, P concentrations in the surface water increased directly after onset of the treatments (after 10 weeks; t = 0). For the high salinity treatments, P concentrations of the surface water showed a strong and significant (P<0.05) decrease immediately after the onset of the treatments (after 10 weeks; t = 0). After this temporary decrease, P concentrations started to increase gradually. As a result, significantly lower P concentrations (p<0.05) were found for the high salinity treatment compared to the low salinity treatment in both sediments at a short term (after 20 weeks; t = 10), and a trend was found when compared to the control treatment (p<0.1) at sediment A. When P mobilisation rates were calculated for the short term, however, no differences among salinity treatments were found (Table 3).

More than 80 weeks after the start of the experiment, P concentrations in the surface water above sediment A were significantly higher (p<0.01) at a high salinity (Fig. 3), which was totally opposite to the short-term effect. Calculated P mobilisation rates were also significantly higher (p<0.05) with a high salinity compared to a low salinity at sediment A. For the control and low salinity treatment, P concentrations in surface water remained low, or even showed a decrease in the long term. At sediment B, however, no change of P in the surface water was found for any of the salinity treatments. The long-term P mobilisation rates with a high salinity were similar to the short-term rates at sediment A, while no long-term P mobilisation was observed for the low salinity and control treatments.

### 3.3 Aerobic versus anaerobic surface water

**Figure 4:**
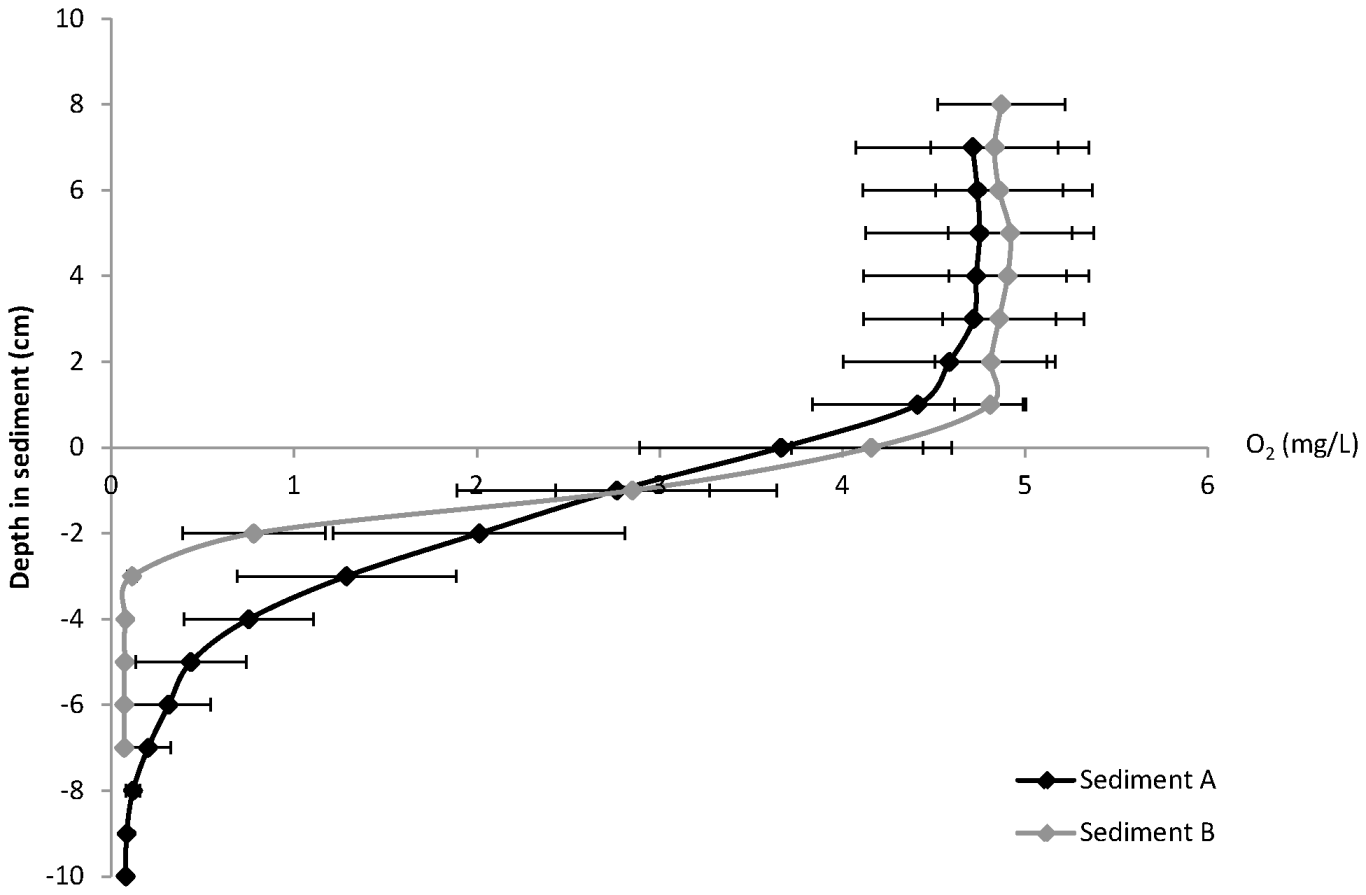
Oxygen (O_2_) concentration (mg L^−1^) profile per mm of both sediments (A and B), at the sediment-water interface (indicated by vertical dotted line) during aerobic and anaerobic conditions.

**Figure 5:**
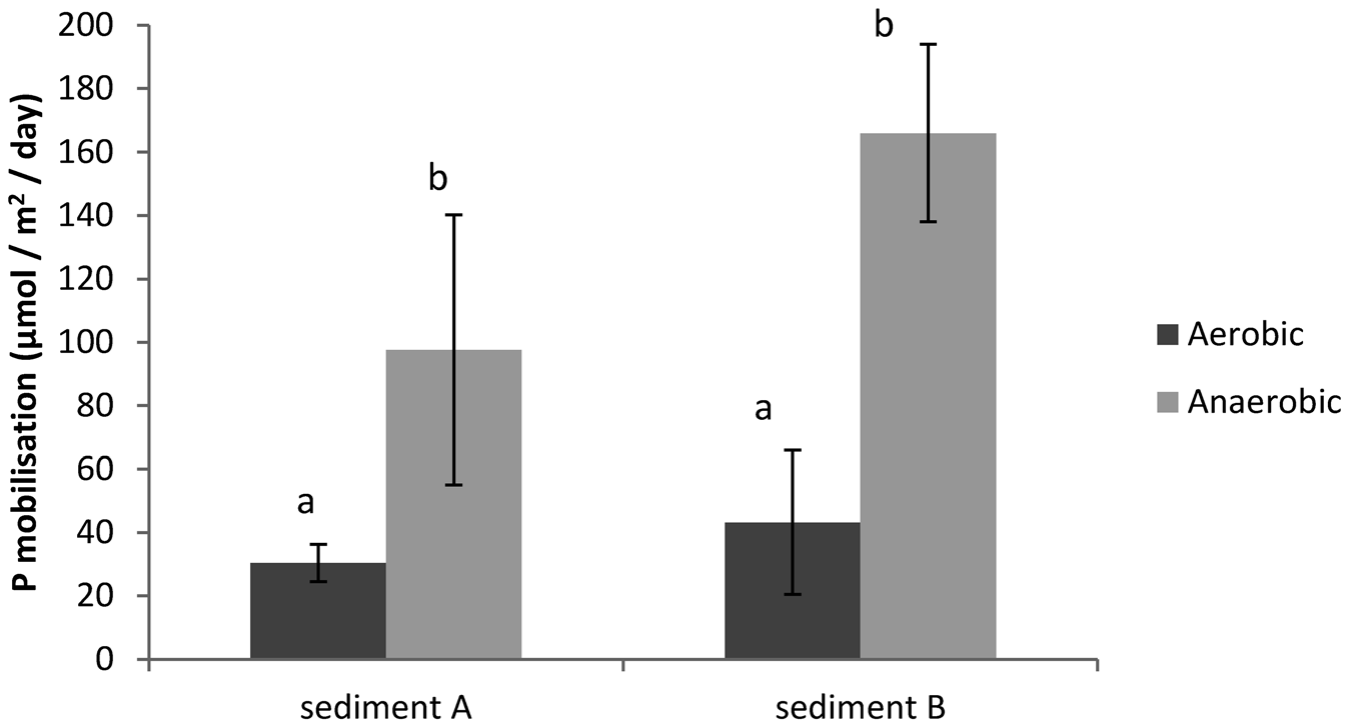
P mobilisation rates (µmol m^−2^ day^−1^) during aerobic and anaerobic conditions for both sediment cores (A and B). Significant differences between treatments are indicated with different letters.

The O_2_ concentration profile (Fig. 4) shows that under aerobic conditions, O_2_ is still available in the sediment to an average depth of 7 mm (sediment A) and 3 mm (sediment B). The cores of both sediment A and B showed a significant (p<0.001) higher mobilisation rate of P during anaerobic conditions (Fig. 5). At sediment A, P mobilisation was on average 3 times higher during anaerobic conditions compared to aerobic conditions, while this was almost 4 times higher at sediment B. These aerobic mobilisation rates were well within range of the short-term mobilisation rates found in the main experiment (control treatment; Table 3).

## Discussion

### 4.1 Short-term effects (0–10 weeks)

#### 4.1.1 P mobilisation

In the short term, no differences in net mobilisation rates of P were found among the different treatments. During this first stagnant period, moderate P mobilisation rates of 7–103 µmol m^−2^ d^−1^ were found that fitted within the range of Geurts *et al*. [19], who found mobilisation rates of 10–150 µmol m^−2^ d^−1^ for sediments of which pore water Fe:P and total sediment Fe:S ratios were <1. Diffusion was most likely the main mechanism driving P release [19], [33], since the sediments used in this experiment were not subjected to bioturbation or resuspension [7], nor to a changed pH or temperature [24]. Moreover, both sediments were characterised by total Fe:S ratios below 0.5 (Table 1), which indicates that most Fe was bound to reduced S [20]. Indeed, dissolved pore water Fe^2+^ concentrations were low in this study (and ranged between 0–20 µmol L^−1^ for both sediments), and showed an even further decrease over time, resulting in very low pore water Fe:P ratios (<0.1) during the entire experimental period.

#### 4.1.2 Salinity effects

Although increased salinity may lead to increased desorption of P from anion exchange sites [10], or by increased S^2-^ production and enhanced mineralisation rates [2], [12], we did not find higher pore water P concentrations in the high salinity treatment. Instead, during the addition of the salinity treatments (between week 6 and 10), P concentrations in the surface water showed a short, strong drop for both sediments. This immediate drop of P observed upon a change of the surface water chemistry strongly points at a chemical, rather than a microbiological, explanation. It can most likely be explained by the co-precipitation of P with Ca^2+^ or CaCO_3_ at the sediment-water interface [4], [9], as Ca^2+^ concentrations directly and strongly increased in both surface and pore water upon the high salinity treatment (0–10 weeks). Accordingly, Suzumura *et al*. [7] found a fast chemical P (im)mobilisation response within minutes, due to adsorption-desorption processes after a changed salinity. Van Dijk *et al*. [34] found a similar immobilisation of P with increased salinity, explained by co-precipitation with Ca^2+^ in the sediment. Degassing of carbon dioxide (CO_2_) and possibly also the presence of microbial mats [35] may well have contributed to the precipitation of CaCO_3_ at the sediment surface, as HCO_3_^-^ concentrations were up to three times higher in pore water than in the surface water. After the initial drop of P, concentrations started to gradually increase, which shows that the short-term overall net P mobilisation to the surface water was higher than its immobilisation due to co-precipitation with Ca^2-^.

### 4.2 Long-term effects (1.5–2 years)

#### 4.2.1 P mobilisation

In contrast to the short-term results, and rather unexpectedly for eutrophic sediments, P mobilisation to the surface water was absent in 5 out of 6 treatments in the longer term (after 80 weeks). This is remarkable, as a strong net diffusive P release in both sediments was expected given the very low pore water Fe^2+^ concentrations and the still very high pore water P concentrations [19]. Although a gradual decrease of P in the pore water of sediment A was observed, concentrations still remained sufficiently high for diffusive P release (>100 µmol L^−1^) [19], [20]. Sediment B even showed a gradual increase of pore water P concentrations during the experiment, without any increase of the P mobilisation to the surface water. Such results can only be explained by assuming that processes preventing net P release at the sediment-water interface become active in the long term, at least under the conditions that were created during our experiment. Possible explanations for this phenomenon are: (1) precipitation of P with Fe^3+^ or Fe(OH)_x_ by the oxidation of the sediment surface [13], [14], (2) storage of P by the microbial community at the sediment surface during aerobic conditions [36], [37], (3) precipitation of P with calcium-minerals [9], [23], although the latter would mainly be expected in the high salinity treatment.

An explanation for the lack of P release in the long term might be the uptake of P by microbial mats growing on top of the sediment [35]–[37]. These mats can develop over time and might also benefit from stable sediment conditions that developed in the experimental set-up. However, our experiment was carried out in the dark, excluding photosynthetically active organisms, and no visible signs of such mats were observed. Nevertheless, the potential role of microbial sequestration of P on the long term cannot be ruled out.

**Figure 6:**
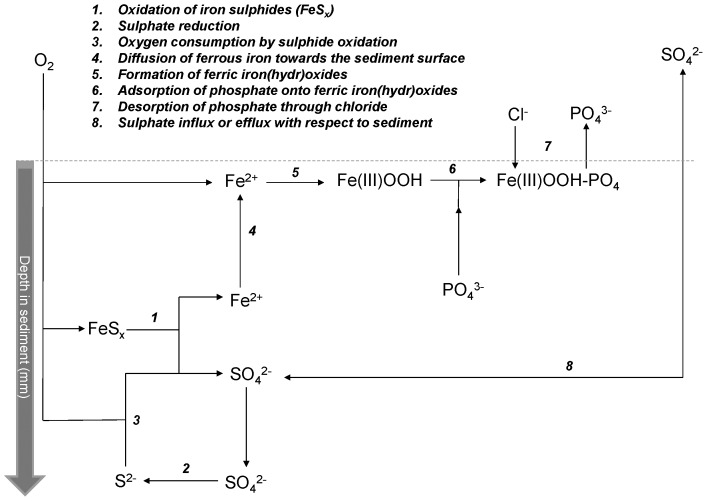
Schematic overview of the proposed mechanism, showing key processes in the upper millimetres of the S-rich, peat sediments involved in P mobilisation. Salinisation leads to an increased SO_4_^2-^ influx, affecting Fe diffusion to the sediment surface, enabling increased P mobilisation in the longer term.

Most likely Fe redox cycling played a dominant role in the absence of P mobilisation, as was also indicated by the strongly increased P release under anaerobic conditions compared to aerobic conditions (Fig. 5). It has been demonstrated that diffusive P release should be prevented under aerobic conditions if pore water Fe:P ratios are relatively high (at least >1) [19], [38], [39]. In our sediments, however, pore water Fe:P ratios were very unfavourable. Nevertheless, oxidation processes might be able to mobilise Fe^2+^ from FeS_x_ at a spatial micro-scale in the sediment surface at relatively low O_2_ levels [40], catalysed by S oxidising microbes [41]. Our O_2_ profiles showed that O_2_ was available in the surface water and in the top millimetres of the sediment. The observed high S mobilisation rates in the low salinity treatment, where no S was added, indeed showed that SO_4_^2-^ is being mobilised from the sediment by the oxidation of FeS_x_. Simultaneously, Fe^2+^ thus becomes available to be oxidised [40], and is able to sequester dissolved P. So the intrusion of O_2_ in reduced sediments may mobilise S bound Fe at a millimetre spatial scale, providing dissolved Fe^2+^ for the formation of ferric Fe(OH)_x_ at the sediment surface (Fig. 6). This mechanism may very well explain the unexpected lack of P release from the sediments in the long term under aerobic conditions.

Our experimental set-up, without sediment disturbance and with relatively low biochemical O_2_ demand (BOD) due to the absence of fresh organic matter input, will certainly have contributed to the long-term outcome of the experiment. Nevertheless, it seems plausible that it took a relatively long time before the sediment surface became sufficiently oxidised, or before the microbial population was sufficiently developed, to completely prevent P mobilisation in the experiment. These results in the longer term may represent field situations with stable non-bioturbated FeS_x_-rich sediments or sediments with stagnant, hypolimnetic water. During anaerobic conditions, P mobilisation was strongly enhanced (Fig. 5), which clearly highlights the importance of O_2_ availability to prevent P release. Field experiments are, therefore, necessary to validate our experimental results and suggested mechanism for the lack of P release from S-rich aquatic sediments.

#### 4.2.2 Salinity effects

For the high salinity treatment, one of the sediments showed an increase of the surface water P concentration also in the long term (80 weeks). In saline or estuarine systems, P is often found to be easily released from soil particles [5], [7], and dissolved P concentrations are usually higher with increasing salinity [6], [8], [39]. At a high salinity, SO_4_^2-^ concentrations increased in both surface water and pore water and a considerable part may be reduced deeper in the sediment, since it was not released to the surface water (Fig. 6). Produced S^2-^ will react with O_2_ and interfere with the oxidation of FeS_x_, or again immobilise Fe^2+^. As expected, the net mobilisation of Fe^2+^ will be less, leading to insufficient formation of ferric Fe(OH)_x_ to prevent the release of P to the surface water [18], [19]. This decoupling of the Fe and P cycle [10] at a micro-scale diminishes the P-binding capacity at the water-sediment interface. In sediment A, O_2_ penetrated deeper into the sediment, suggesting that less O_2_ was consumed, less FeS_x_ was oxidised, and less Fe^2+^ was mobilised. This may partly explain the long-term release of P from sediment A in the high salinity treatment. Desorption of P from ferric Fe(OH)_x_ due to the high Cl concentrations [10], [22] might have increased this effect.

### 4.3 Implications for water management

Although the mobilisation of P from the S-rich and relatively Fe-poor sediments (typical for coastal wetlands) was mainly driven by diffusion, the build-up of a stable oxidised sediment surface may have prevented the release of P under the experimental conditions. We hypothesise that the oxidation of FeS_x_ in the sediment surface delivers the Fe^2+^ necessary for the precipitation of P at the sediment-water interface (Fig. 6). Disturbance of the sediment-water interface due to wind, ebullition of gases from the sediment, and bioturbation can, however, prevent this build-up of a protective Fe-rich sediment surface and potentially increase the release of P [9], [33]. Although such processes might also mix the sediment surface with O_2_ and have an opposite effect. Moreover, our results indicate that an increased salinity may lead to a long-term P release, probably by interfering with the Fe^2+^ mobilisation due to increased SO_4_^2-^ reduction rates in the anaerobic sediment. They also point out that sediments may react differently upon increased salinity. Therefore, O_2_ and BOD, but also the actual concentration of SO_4_^2-^ play a key role in the mobilisation of P from FeS_x_-rich sediments. This might have important implications for water management and nature management of eutrophic peatlands in relation to salinisation.

More research, especially field measurements, is necessary to further confirm the experimental results we found for these FeS_x_-rich sediments. Our experiment was carried out at 15°C and without the continuous input of reactive organic material. Warmer conditions, e.g. during warm episodes in summer will lead to increased mineralisation rates, and also to higher O_2_ consumption rates and lower solubility of O_2_. Especially when there is a high input of reactive organic matter, this will lead to strongly decreased O_2_ concentrations in the surface water, which may prevent adequate oxidation of the sediment surface. Under such conditions this biogeochemical mechanism is expected to fail, leading to strong P mobilisation from the sediment as was shown in this study and also found by Smolders *et al.* [12]. As a result, floating-leaved species, or floating beds of algae or cyanobacteria may develop, which will further decrease the O_2_ concentrations in the surface water and enhance sediment P mobilisation. This explains why FeS_x_-rich sediments that show very high dissolved P concentrations and low dissolved Fe^2+^ concentrations tend to show a high P release mainly in summer, which has important implications for water management.

## Conclusions

- Low pore water Fe:P ratios indicated a decoupling of the Fe and P cycle. Although these FeS_x_-rich sediments were expected to release significant amounts of P by diffusion, this only seemed to be the case in the short term under aerobic conditions.
- Increased salinity led to co-precipitation of P with Ca^2+^ in the short term, lowering actual P concentrations. However, short-term P mobilisation rates were found to be similar for all treatments, regardless of salinity.
- Our experimental results suggest that the classic theoretical framework of oxidative conditions in the surface water that prevent P release from the sediment, may also hold in sediments showing unfavourable total Fe:S ratios but high FeS_x_ concentrations. In our FeS_x_-rich, eutrophic sediments, typical for coastal wetlands, O_2_ availability still seemed to be the most important determinant of sediment P release, at least under stable sediment conditions.
- We suggest a controlling mechanism in which the partial oxidation of FeS_x_ mobilises sufficient Fe^2+^ at micro-scale for the precipitation of P at the sediment-water interface.
- Next to O_2_, SO_4_^2-^ plays a key role in P mobilisation, as high concentrations may counteract the oxidising effect by immobilising Fe^2+^. In the longer term, an increased salinity may, as a result, led to P mobilisation despite oxidation of the sediment surface.
