# Correction: New Insights into Phosphorus Mobilisation from Sulphur-Rich Sediments: Time-Dependent Effects of Salinisation

**DOI:** 10.1371/journal.pone.0118387

**Published:** 2015-03-30

**Authors:** The PLOS ONE Staff

There is an error in Fig. 4, “Oxygen (O_2_) concentration (mg L^−1^) profile per mm of both sediments (A and B), at the sediment-water interface (indicated by vertical dotted line) during aerobic and anaerobic conditions.” The title of the y-axis should read: Depth in sediment (mm). Please see the corrected Fig. 4 here.

**Fig 4:**
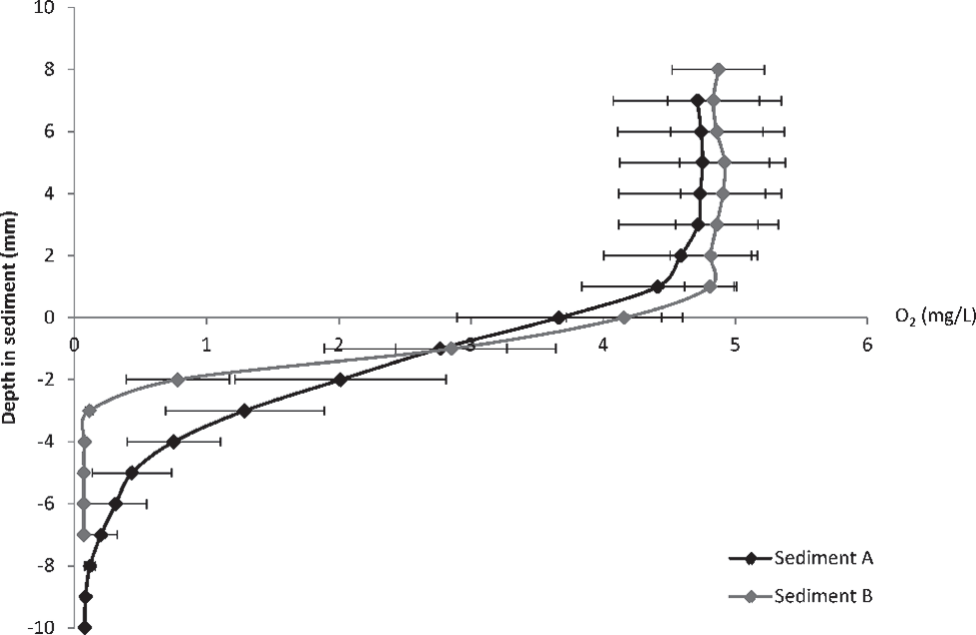
Oxygen (O_2_) concentration (mg L^−1^) profile per mm of both sediments (A and B), at the sediment-water interface (indicated by vertical dotted line) during aerobic and anaerobic conditions.
